# Evaluating future risk of NAFLD in adolescents: a prediction and decision curve analysis

**DOI:** 10.1186/s12876-022-02401-y

**Published:** 2022-06-30

**Authors:** Kushala W. M. Abeysekera, James G. Orr, Fiona H. Gordon, Laura D. Howe, Julian Hamilton-Shield, Jon Heron, Matthew Hickman

**Affiliations:** 1 Population Health Science, Bristol Medical School, University of Bristol, Bristol, UK; 2 Department of Liver Medicine, University Hospitals Bristol and Weston NHS Foundation Trust, Bristol, UK; 3 MRC Integrative Epidemiology Unit, University of Bristol, Bristol, UK; 4 NIHR Bristol Biomedical Research Centre, Nutrition Theme, University Hospitals Bristol and Weston NHS Foundation Trust & University of Bristol, Bristol, UK

**Keywords:** ALSPAC (Avon Longitudinal Study of Parents and Children), Body composition, NAFLD (nonalcoholc fatty liver disease), Obesity, Young adults

## Abstract

**Background:**

Non-alcoholic fatty liver disease (NAFLD) is the commonest liver condition in the western world and is directly linked to obesity and the metabolic syndrome. Elevated body mass index is regarded as a major risk factor of NAFL (steatosis) and NAFLD fibrosis. Using data from the Avon Longitudinal Study of Parents and Children (ALSPAC), we sought to investigate whether other variables from adolescence could improve prediction of future NAFL and NAFLD fibrosis risk at 24 years, above BMI and sex.

**Methods:**

Aged 24 years, 4018 ALSPAC participants had transient elastography (TE) and controlled attenuation parameter (CAP) measurement using Echosens 502 Touch. 513 participants with harmful alcohol consumption were excluded. Logistic regression models examined which variables measured at 17 years were predictive of NAFL and NAFLD fibrosis in young adults. Predictors included sex, BMI, central adiposity, lipid profile, blood pressure, liver function tests, homeostatic model assessment for insulin resistance (HOMA-IR), and ultrasound defined NAFL at 17 years (when examining fibrosis outcomes). A model including all these variables was termed “routine clinical measures”. Models were compared using area under the receiver operator curve (AUROC) and Bayesian Information Criterion (BIC), analysis, which penalises model complexity. Models were tested in all participants and those with overweight or obese standardised BMIs (BMI SDS) centiles at the 17-year time point. A decision curve analysis (DCA) was performed to evaluate the clinical utility of models in overweight and obese adolescents predicting NAFLD fibrosis at a threshold probability of 0.1.

**Results:**

The “routine clinical measures” model had the highest AUROC for predicting NAFL in all adolescent participants (AUROC 0.79 [SD 0.00]) and those with an overweight/obese BMI SDS centile (AUROC 0.77 [SD 0.01]). According to BIC analysis, insulin resistance was the best predictor of NAFL in all adolescents, whilst central adiposity was the best predictor in those with an overweight/obese BMI SDS centile. The “routine clinical measures” model also had the highest AUROC for predicting NAFLD fibrosis in all adolescent participants (AUROC 0.78 [SD 0.02]) and participants with an overweight/obese BMI SDS centile (AUROC 0.84 [SD 0.03]). However, following BIC analysis, BMI was the best predictor of NAFLD fibrosis in all adolescents including those with an overweight/obese BMI SDS centile. A decision curve analysis examining overweight/obese adolescent participants showed the model that had the greatest net benefit for increased NAFLD fibrosis detection, above a treat all overweight and obese adolescents’ assumption, was the “routine clinical measures” model. However, the net benefit was marginal (0.0054 [0.0034–0.0075]).

**Conclusion:**

In adolescents, routine clinical measures were not superior to central adiposity and BMI at predicting NAFL and NAFLD fibrosis respectively in young adulthood. Additional routine clinical measurements do provide incremental benefit in detecting true positive fibrosis cases, but the benefit is small. Thus, to reduce morbidity and mortality associated with NASH cirrhosis in adults, the ultimate end point of NAFLD, the focus must be on obesity management at a population level.

**Supplementary Information:**

The online version contains supplementary material available at 10.1186/s12876-022-02401-y.

## Background

Non-alcoholic fatty liver disease (NAFLD) is the commonest liver disease in the Western world. Modelling from multiple countries have demonstrated rapidly rising rates of NAFLD cirrhosis in the general population. In Wales (UK), Pembroke et al. demonstrated NAFLD incidence had increased tenfold between 1999 and 2019, using Welsh registry mortality data from the Office of National Statistics [1]. Using modelling data from Canada, based on current trends, 73% of all new diagnoses of cirrhosis will be linked to NAFLD by 2040, without intervention [2]. Markov modelling based on existing UK prevalence of obesity and type 2 diabetes mellitus predicts the number of patients with advanced NAFLD fibrosis and cirrhosis to double from approximately 500,000 to 1 million by 2030 [3].

Whilst not commonly causing overt morbidity in the paediatric setting, NAFLD is present even in early life. A systematic review of NAFLD prevalence, among other co-morbidities, in 6–18 years, found children and adolescents to have a 6 to 26 times higher prevalence of NAFLD when overweight or obese respectively [4].

NAFLD, obesity, insulin resistance and cardiovascular disease are linked under the overarching term of the metabolic syndrome. The leading cause of death in NAFLD remains cardiovascular disease, whilst fibrosis, not steatosis (or NAFL), is associated with increased liver-related event and mortality [5–7]. Therefore, there remains ongoing interest in how cardiometabolic markers such as lipid profiles, fasting lipid profiles, glucose and insulin shape the evolution of NAFLD.

The Avon Longitudinal Study of Parents and Children (ALSPAC) UK birth cohort has examined its participants for NAFLD at two time points, when they are were 17.8 years and 24.0 years, utilising ultrasound and transient elastography respectively. The prevalence of NAFLD was 2.5% at age 17 versus 20.7% at age 24, whilst at age 24 years 2.4% of the cohort had NAFLD fibrosis [8]. Furthermore, using ALSPAC NAFLD prevalence data at 17.8 years, Anderson et al. examined weight trajectories through infancy, childhood, and the associated risk of NAFLD in adolescence. Weight change in childhood at multiple time points was consistently associated with NAFLD development in adolescence, however when adjusted for fat mass at 17.8 years, the association attenuated [9].

Given NAFLD is largely driven by adiposity and elevated body mass index (BMI), the aim of this study, utilising existing ALSPAC data, was to elucidate if cardiometabolic markers and liver function tests performed in adolescence improve our prediction of the future risk of NAFL and NAFLD fibrosis in young adulthood, above and beyond BMI. To further understand the clinical utility of these variables, a decision curve analysis was performed to evaluate their net benefit of testing adolescents with overweight and obese BMI centiles to predict NAFLD fibrosis and target interventions.

## Methods

### Study population

The Avon Longitudinal Study of Parents and Children (ALSPAC) is a prospective birth cohort study from southwest England [10]. The study website contains details of all available data through a fully searchable data dictionary and variable search tool (www.bristol.ac.uk/alspac/researchers/our-data). Briefly, ALSPAC invited pregnant women in Avon, UK with expected delivery dates between April 1, 1991 and December 31, 1992 into the cohort [11]. The initial number of pregnancies enrolled is 14,541. Of these initial pregnancies, there was a total of 14,676 foetuses, resulting in 14,062 live births and 13,988 children who were alive at 1 year of age [10].

When the oldest children were approximately 7 years of age, an attempt was made to bolster the initial sample with eligible cases who had failed to join the study originally. Following 3 further phases of recruitment, this resulted in an additional 913 children being enrolled. The total sample size for analyses using any data collected after the age of seven is therefore 15,454 pregnancies, resulting in 15,589 foetuses. Of these 14,901 were alive at 1 year of age [11].

ALSPAC data from two clinic time points has been included. The first was the Teen Focus 4 (TF4) clinic held between December 2008 and June 2011. This was attended by 5081 participants (mean age 17.8 years). The second, and most recent, was Focus @ 24 + clinic held between June 2015 and October 2017. This was attended by 4018 participants (mean age 24.0 years) [8].

Study data were collected and managed using REDCap electronic data capture tools hosted at University of Bristol [12]. In line with ALSPAC confidentiality policy, any analysed groups with less than five participants are expressed as n = “< 5”. This figure when expressed can include zero.

In the F@24+ clinic no one was reported to have viral hepatitis or taking nucleoside analogues/direct-acting antivirals. In the F@24+ clinic, there were less than 5 participants identified with: autoimmune hepatitis on azathioprine, or autoimmune hepatitis/primary sclerosing cholangitis overlap syndrome on prednisolone, mycophenolate mofetil, ursodeoxycholic acid, who were not removed as this was a general population study [8].

### Outcomes

Prior to Focus@24+, participants were fasted for a minimum of 6 h or overnight before blood tests and subsequent liver imaging.

Imaging. In the F@24+ clinic participants were assessed with transient elastography (TE; FibroScan®, Echosens 502 Touch®, Echosens, Paris) and controlled attenuation parameter (CAP). These are a standardised non-invasive measure of fibrosis and quantifying steatosis in NAFLD [13]. CAP score cut off values for different grades of steatosis for both groups were derived from a meta-analysis on CAP technology [14]. Ten valid readings within the range of 100–400 dB/m were required to derive a CAP score. Ten valid readings and an interquartile range/median (IQR/M) ratio < 30% were required to interpret a liver stiffness measurement (LSM).

**Fig. 1:**
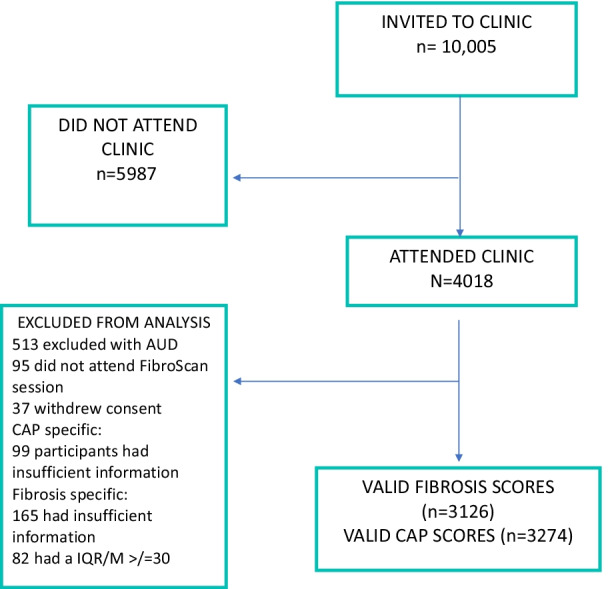
Participant flow chart in Focus@24 clinic

744 participants were excluded from the final analysis of steatosis or NAFL. 513 participants attending the F@24+ clinic had evidence of Diagnostic and Statistical Manual of Mental Disorders-5 (DSM-V) criteria for alcohol use disorder (AUD) were excluded from analysis [15]. 95 participants did not attend the TE session. Of those who did, 99 had insufficient information e.g., did not have 10 valid CAP measurements. Finally, 37 participants withdrew consent to continue being part of ALSPAC. Thus, 3274 participants were included in this study with outcome steatosis or NAFL. When assessing NAFLD fibrosis outcomes, in addition to individuals who did not attend the TE clinic, withdrew consent, or had harmful alcohol consumption, individuals with an IQR/M ≥ 30 were excluded. This left 3126 participants remaining with fibrosis outcomes (Fig. 1) [16].

### Potential predictors

#### Anthropometry and blood pressure

On arrival to the focus clinics participants had their standing height (cm), weight (kg) and waist circumference (cm) documented. This facilitated body mass index (BMI) and waist circumference-to-height (WCHt) calculation. WCHt was used as a marker of central adiposity [17–19]. Standing height was measured using a Harpenden wall-mounted stadiometer. Weight was measured using Tanita TBF-401A electronic body composition scales (or electronic bathroom scales, if the participant had a pacemaker). Circumferences were measured using Seca 201 body tension tape and were repeated twice for accuracy. Heights and circumferences were measured to the nearest millimetre, while weight was measured to the nearest 0.1 kg. Fieldworker variation was recorded in the Focus@24 clinic for anthropometric measurements: for weight, % variation amongst fieldworkers was 0.5% (*p* = 0.392); height 0.8% (*p* = 0.056); mean waist circumference 3.3% (*p* < 0.0001). Systolic and diastolic blood pressure (BP) measurements were performed on each arm and a mean value was documented.

#### Serology

Blood samples were immediately centrifuged and frozen at − 80 °C. Samples were analysed through standard clinical chemistry assays as previously described [20]. Fasted blood tests were performed at Teen Focus Clinic 4 (TF4) and Focus@24 clinic. Participants were not fasted at the Focus@9 clinic. Samples were analysed including liver blood tests: alanine transaminase (ALT), aspartate aminotransferase (AST) and γ-glutamyl transferase (GGT). Lipid profiles were taken including cholesterol, triglycerides, low-density lipoprotein (LDL-C), very low-density lipoprotein (VLDL-C) and high-density lipoprotein (HDL-C). Glucose and insulin levels were also sent and used to calculate the homeostasis model assessment for insulin resistance (HOMA-IR), using the equation HOMA-IR score = (Fasting insulin [µU/ml] × Fasting glucose [mmol/l])/22·5 [21].

#### Imaging

Steatosis was examined in the Teen Focus 4 clinic with upper abdominal ultrasound in a random subset of 1887 participants. This was performed by one of four trained sonographers using a Siemens Acuson S2000 USS system [22]. Echogenicity, as a marker of liver fat, was assessed based on established protocols and graded as present/absent/or uncertain [23].

### Statistical analysis

The purpose of the analysis was to examine how different models predicted outcomes of NAFL (defined as CAP score ≥ 248 dB/m equivalent to S1 steatosis) and NAFLD fibrosis ((defined as a LSM > 7.9 kPa equivalent to ≥ F2 fibrosis) at 24 years at two time points: the 24-year and 17-years (see Tables 1, 2). These models were estimated in all participants, representing the general population, and then in participants with a BMI ≥ 25 kg/m^2^ at the 24-year time point or weight centiles consistent with an overweight/obese standardised BMI (BMI SDS) at the 17-year time point. The rationale for this was to test if the models we were evaluating would be of greater benefit in individuals that may attend weight management services.

**Table 1:** Summary of models considered with NAFL outcomes at 24 years

Model	Model name	Model components
1	Sex	Sex
2	BMI	Sex, BMI (log)
3	Central adiposity	Sex, BMI (log), WCHt ratio
4	Dyslipidaemia	Sex, BMI (log), triglycerides(log), cholesterol, LDL-C, VLDL-C, HDL-C
5	Insulin resistance	Sex, BMI (log), HOMA-IR
6	Hypertension	Sex, BMI (log), systolic BP, diastolic BP
7	Cardiometabolic	Sex, BMI (log), triglycerides(log), cholesterol, LDL-C, VLDL-C, HDL-C, HOMA-IR, systolic BP, diastolic BP
8	Liver enzymes	Sex, BMI (log), ALT (log), AST (log), GGT (log)
9	Routine clinical measures	Sex, BMI (log), WCHt ratio, triglycerides(log), cholesterol, LDL-C, VLDL-C, HDL-C, HOMA-IR, systolic BP, diastolic BP, ALT (log), AST (log), GGT (log)

**Table 2:** Summary of models considered with NAFLD fibrosis outcomes at 24 years

Model	Model name	Model components
1	Sex	Sex
2	BMI	Sex, BMI (log)
3	Central adiposity	Sex, BMI (log), WCHt ratio
4	Steatosis	Sex, BMI (log), steatosis^a^
5	Dyslipidaemia	Sex, BMI (log), triglycerides(log), cholesterol, LDL-C, VLDL-C, HDL-C
6	Insulin resistance	Sex, BMI (log), HOMA-IR
7	Hypertension	Sex, BMI (log), systolic BP, diastolic BP
8	Cardiometabolic	Sex, BMI (log), triglycerides(log), cholesterol, LDL-C, VLDL-C, HDL-C, HOMA-IR, systolic BP, diastolic BP
9	Liver enzymes	Sex, BMI (log), ALT (log), AST (log), GGT (log)
10	Cardiometabolic and liver enzymes	Sex, BMI (log), WCHt ratio, triglycerides(log), cholesterol, LDL-C, VLDL-C, HDL-C, HOMA-IR, systolic BP, diastolic BP, ALT (log), AST (log), GGT (log)
11	Routine clinical measures	Sex, BMI (log), WCHt ratio, steatosis^a^, triglycerides(log), cholesterol, LDL-C, VLDL-C, HDL-C, HOMA-IR, systolic BP, diastolic BP, ALT (log), AST (log), GGT (log)

^a^USS defined steatosis at 17-year time point and CAP defined steatosis at 24-year time point

All models tested included sex and BMI. Additional predictors considered included central adiposity (based on WCHt) lipid profile, blood pressure, liver function tests, homeostatic model assessment for insulin resistance (HOMA-IR). When examining NAFLD fibrosis outcome at 24 years specifically, the additional predictors of steatosis at 17 years (based on ultrasound) and 24 years (based on CAP score) were included. Model names are listed in Tables 1 and 2. A “routine clinical measures” model included all the predictors tested, reflecting an assessment of a patient for NAFLD in clinic.

Models were incrementally augmented with different variables auditioned sequentially. The predictive ability of each model was compared using area under the receiver operator curve (AUROC) and the Bayesian Information Criterion (BIC) analyses. The model with the lowest BIC was considered the best model. BIC was used in preference of AUROC as it penalises model complexity and seeks the most parsimonious model, whilst AUROC would be expected to rise as the number of variables inputted into the model increases.

A power calculation for the binary outcomes of steatosis and fibrosis were performed to ascertain if the sample size within our cohort was large enough to develop a model that could be used in other populations with NAFL and NAFLD fibrosis. We opted to move away from the rule of ten events per variable in preference of events per candidate predictor parameter [24] when considering our binary outcome of NAFL and NAFLD fibrosis. The maximum number of candidate predictors for NAFL outcomes was 14 in model 9 (see Table 1). Based on a NAFL events fraction of 0.2 (20% NAFL population prevalence) and an estimator error of 0.05, the minimum required total sample size was 860. The maximum number of candidate predictors for NAFLD fibrosis outcomes was 15 in model 11 (see Table 2). Based on a NAFLD fibrosis events fraction of 0.02 (2.7% based on prevalence fibrosis prevalence work within the ALSPAC cohort) and an estimation error of 0.05, the minimum required total sample size was 1800.

To explore clinical applicability of using these prediction models, we used a decision curve analysis to explore which model would provide the greatest net benefit in a clinical scenario [25]. We chose the scenario of clinicians reviewing an adolescent patient who was overweight or obese in a paediatric clinic or primary care, and which model would best trigger an intervention to prevent NAFLD fibrosis in young adulthood. Net benefit combines the number of true positives and false positive and is obtained by dividing the net true positives by the sample size. We used a low-risk threshold of 10% (0.1) when interrogating the decision curve analyses, as we deemed it acceptable to intervene on 10 patients with an overweight/obese centile at 17 years to prevent 1 patient developing NAFLD fibrosis by the age of 24 years. We can use a low threshold as interventions, surrounding weight management, are non-invasive and have minimal complications compared to liver biopsy for example.

### Dealing with missing data

In this study we only included participants with complete CAP data (n = 3274) or complete liver stiffness measurement data (n = 3126) and imputed missing exposure and confounder data. Correlation matrices were performed at each time point and across time points to identify auxiliary variable candidates to be utilised for multiple imputation at for each model within each clinic.

Missing data was assumed to be dependent on observed data from previous time points and missing at random. 100 data sets were imputed using auxiliary variables up to the total number of valid CAP measurements. Multivariable regression models were then performed across imputed datasets using the ‘mi estimate’ command which fits a model to each of the imputed data set and pools individual results using Rubin’s combination rules [26]. Statistical analysis was performed using Stata MP 17·1.

## Results

### NAFL outcomes at 24 years

In total 3274 participants (63.9% female) had valid CAP measurements that were included in the analysis (see Fig. 1). Mean CAP score was 208.6 dB/m (SD 53.8). Across all 9 prediction models interrogated, 1459 and 2568 participants had complete data available at the 17-year and 24-year clinic respectively. Summary data on variables included in predictive models are shown in Table 3.

**Table 3:** Summary data on variables included in prediction models in Teen Focus 4 and Focus at 24 years clinic

Total number of valid CAP measurements = 3274		Teen Focus 4 Clinic (mean age 17.8 years)			Focus@24+ Clinic (mean age 24.0 years)		
		Males	Female		Male	Female	
BMI (kg/m^2^)	Median (IQR)	21.7 (20.0–24.1) n = 898	21.9 (20.0–24.5) n = 1581	*p* = 0.11	24.2 (21.8–26.9) n = 1176	23.4 (21.2–27.0) n = 2067	*p* = 0.002
Waist circumference-to-height ratio	Median (IQR)	0.43 (0.41–0.45) n = 737	0.45 (0.42–0.49) n = 1250	*p* < 0.0001	0.47 (0.43–0.51) n = 1173	0.45 (0.42–0.51) n = 2061	*p* < 0.0001
Triglycerides (< 1.7 mmol/l)	Median (IQR)	0.7 (0.6–0.9) n = 685	0.7 (0.6–1.0) n = 969	*p* = 0.11	0.9 (0.7–1.2) n = 1011	0.8 (0.6–1.1) n = 1647	*p* < 0.0001
Cholesterol (< 5·2mmmol/l)	Mean (SD)	3.6 (0.6) n = 685	3.9 (0.7) n = 969	*p* < 0.0001	4.4 (0.8) n = 1012	4.5 (0.8) n = 1647	*p* < 0.0001
LDL-C (mmol/l)	Mean (SD)	2.0 (0.6) n = 685	2.2 (0.6) n = 969	*p* < 0.0001	2.5 (0.8) n = 1010	2.4 (0.8) n = 1647	*p* = 0.22
VLDL-C (mmol/l)	Median (IQR)	0.3 (0.3–0.4) n = 685	0.3 (0.3–0.4) n = 969	*p* = 0.06	0.4 (0.3–0.6) n = 1010	0.4 (0.3–0.5) n = 1647	*p* < 0.0001
HDL-C (> 1·45 mmol/l)	Mean (SD)	1.2 (0.3) n = 685	1.3 (0.3) n = 969	*p* < 0.0001	1.4 (0.4) n = 1012	1.6 (0.4) n = 1647	*p* < 0.0001
HOMA-IR (< 1·68)	Median (IQR)	1.3 (1.0–2.0) n = 675	1.6 (1.1–2.2) n = 947	*p* < 0.0001	1.8 (1.2–2.5) n = 1012	1.8 (1.2–2.7) n = 1647	*p* = 0.04
Systolic blood pressure (mmHg)	Mean (SD)	124.1 (10.1) n = 860	113.9 (9.1) n = 1482	*p* < 0.0001	122.9 (10.8) n = 1179	111.6 (9.6) n = 2084	*p* < 0.0001
Diastolic blood pressure (mmHg)	Mean (SD)	63.0 (6.4) n = 860	64.4 (6.4) n = 1482	*p* < 0.0001	67.6 (8.1) n = 1179	66.4 (7.8) n = 2084	*p* = 0.0001
ALT (10–35 U/l)	Median (IQR)	16.4 (12.8–21.6) n = 678	14.0 (11.3–17.8) n = 962	*p* < 0.0001	26.4 (19.6–37.0) n = 1012	17.4 (13.7–23.3) n = 1645	*p* < 0.0001
AST (10–35 U/l)	Median (IQR)	20.6 (17.7–24.9) n = 678	18.5 (16.2–21.6) n = 962	*p* < 0.0001	27.4 (22.8–33.1) n = 1012	22.6 (19.6–26.6) n = 1645	*p* < 0.0001
GGT (< 40 U/l)	Median (IQR)	18.0 (15.0–23.0) n = 678	15.0 (12.0–18.0) n = 961	*p* < 0.0001	18.0 (14.0–26.0) n = 1012	14.0 (11.0–19.0) n = 1647	*p* < 0.0001

(See Table 4) The models with the highest AUROC amongst all participants aged 24 years was the “RCM” model (model 9 AUROC 0.86 [SD 0.00]; BIC 2434.15 [SD 10.96]). However, the lowest BIC at this time point was the “cardiometabolic” model (model 7; BIC 2418.27 [SD 10.53]; AUROC 0.85 [SD0.00]). In all participants at the 17-year clinic timepoint, the highest AUROCs were the “insulin resistance” and “RCM” model jointly (model 5 AUROC 0.79 [SD 0.01] and BIC 2799.42 [SD23.45]; model 9 AUROC 0.79 [SD 0.00] and BIC 2833.75 [SD 24.27]). The model with the lowest BIC was also the “insulin resistance” model.

**Table 4:** BIC and AUROC at 17 and 24-year timepoint assessing the outcome of NAFL at 24 years in all participants and overweight participants (at 17-year and 24-year time point). Imputed results presented

Model	Model components	17-year model (n = 3274)				24-year model (n = 3274)			
		All participants (n = 3274)		Participants with a BMI > overweight BMI SDS centile (n = 1322)		All participants (n = 3274)		Participants with BMI > 25 kg/m^2^ (n = 1239)	
		BIC Mean (SD)	AUROC Mean (SD)	BIC Mean (SD)	AUROC Mean (SD)	BIC Mean (SD)	AUROC Mean (SD)	BIC Mean (SD)	AUROC Mean (SD)
1. Sex	Sex	3322.6 (0)	0.56 (0)	1640.3 (0.0)	0.54 (0.0)	3322.6 (0)	0.56 (0)	1692.7 (0.0)	0.55 (0)
2. BMI	Sex, BMI (log)	2830.3 (23.0)	0.77 (0.0)	1457.4 (22.2)	0.74 (0.0)	2483.3 (3.1)	0.84 (0.00)	1470.2 (0.0)	0.74 (0.0)
3. Central adiposity	Sex, BMI (log), WCHt ratio	2849.1 (14.5)	0.77 (0.0)	1441.7 (17.9)	0.75 (0.0)	2448.6 (2.7)	0.85 (0.00)	1397.3 (3.6)	0.78 (0.0)
4. Dyslipidaemia	Sex, BMI (log), triglycerides(log), cholesterol, LDL-C, VLDL-C, HDL-C	2852.2 (24.1)	0.77 (0.0)	1477.4 (24.6)	0.74 (0.0)	2469.0 (7.1)	0.84 (0.00)	1463.6 (5.8)	0.76 (0.0)
5. Insulin resistance	Sex, BMI (log), HOMA-IR	2799.4 (23.4)	0.79 (0.0)	1456.2 (22.1)	0.74 (0.01)	2419.2 (9.0)	0.85 (0.00)	1396.5(10.3)	0.78 (0.0)
6. Hypertension	Sex, BMI (log), systolic BP, diastolic BP	2826.1 (22.9)	0.77 (0.0)	1452.7 (24.0)	0.74 (0.01)	2468.7 (3.1)	0.84 (0.00)	1463.3 (0.8)	0.76 (0.0)
7.Cardiometabolic	Sex, BMI (log), triglycerides(log), cholesterol, LDL-C, VLDL-C, HDL-C, HOMA-IR, systolic BP, diastolic BP	2832.9 (23.9)	0.78 (0.0)	1477.2 (24.8)	0.75 (0.01)	2418.3 (10.5)	0.85 (0.00)	1414.1 (9.8)	0.79 (0.0)
8. Liver enzymes	Sex, BMI (log), ALT (log), AST (log), GGT (log)	2818.2 (23.9)	0.78 (0.0)	1466.3 (23.8)	0.74 (0.01)	2479.5 (6.5)	0.84 (0.00)	1468.5 (6.1)	0.75 (0.0)
9. Routine clinical measures	Sex, BMI (log), WCHt ratio, triglycerides(log), cholesterol, LDL-C, VLDL-C, HDL-C, HOMA-IR, systolic BP, diastolic BP, ALT (log), AST (log), GGT (log)	2851.9 (21.5)	0.79 (0.0)	1470.4 (23.9)	0.77 (0.01)	2421.0 (9.2)	0.86 (0.00)	1421.5 (10.4)	0.80 (0.0)

(See Table 4) 1322 participants (65.3% female) and 1239 participants (60.0% female) with valid CAP measurements had an ≥ overweight BMI SDS centile at the 17-year clinic and BMI ≥ 25 kg/m^2^ in the 24-year clinic respectively. In all participants the 17-year and 24-year time points, the highest AUROC was for the “RCM” model (model 9; AUROC 0.79 [SD 0.01] and 0.81 [SD 0.00] respectively). At the 17-year time point the “central adiposity” model had the lowest BIC (BIC 1441.74 [SD 17.89]; AUROC 0.75 [SD0.01]). Whilst at the 24-year time point the “insulin resistance” model had the lowest BIC (model 5; BIC 1396.49 [SD 10.32]),

### NAFLD fibrosis outcomes at 24 years

(See Table 5) 3126 participants had valid LSMs (63.3% female). Mean LSM was 4.7 kPa (SD 1.5). At the 24-year timepoint, the highest AUROC were the “cardiometabolic and liver enzyme” (model 10 AUROC 0.73 [SD 0.01]; BIC 776.10 [SD 4.63]) and “RCM” models (model 11 AUROC 0.73 [SD 0.01]; BIC 783.00 [SD 4.60]). However, the lowest BICs were the “sex only” (BIC 724.31 [SD 0.00]; AUROC 0.57 [SD0.00]) and “BMI” models (BIC 725.31; SD 0.07; AUROC 0.62 [SD 0.00]). At the 17-year time point, the “RCM” models had the highest AUROC (model 11 AUROC 0.78 [SD 0.02]; BIC 764.38 [SD 10.06). The model with the lowest BIC at this time point was the “BMI” model (model 2 BIC 710.39 [SD 0.34]; AUROC 0.67 [SD 0.01]).

**Table 5:** BIC and AUROC at 17-year and 24-year timepoint assessing the outcome of NAFLD Fibrosis at 24 years in all participants and overweight participants (at 17-year and 24-year time point). Imputed results presented

Model	Model components	17-year model				24-year model			
		All participants (n = 3126)		Participants with a BMI > overweight BMI SDS centile (n = 1160)		All participants (n = 3126)		Participants with BMI > 25 kg/m^2^ (n = 1163)	
		BIC Mean (SD)	AUROC Mean (SD)	BIC Mean (SD)	AUROC Mean (SD)	BIC Mean (SD)	AUROC Mean (SD)	BIC Mean (SD)	AUROC Mean (SD)
1. Sex	Sex	724.3 (0)	0.57 (0)	354.3 (0)	0.54 (0)	724.3 (0)	0.57 (0)	313.4 (0)	0.54 (0)
2. BMI	Sex, BMI (log)	710.4 (3.7)	0.67 (0.01)	350.0 (3.9)	0.64 (0.02)	725.3 (0.1)	0.62 (0.00)	312.4 (0.0)	0.62 (0.00)
3. Central adiposity	Sex, BMI (log), WCHt ratio	713.6 (3.0)	0.68 (0.01)	352.6 (3.8)	0.67 (0.02)	730.8 (0.1)	0.64 (0.00)	319.5 (0.0)	0.62 (0.00)
4. Steatosis	Sex, BMI (log), steatosis ^†^	714.4 (7.4)	0.68 (0.01)	350.5^†^ (7.6)	0.67^†^ (0.04)	733.1 (0.1)	0.62 (0.00)	319.4^‡^ (0.0)	0.62 ^‡^ (0.00)
5. Dyslipidaemia	Sex, BMI (log), triglycerides(log), cholesterol, LDL-C, VLDL-C, HDL-C	733.5 (7.1)	0.71 (0.02)	364.8 (11.0)	0.74 (0.04)	748.8 (2.3)	0.64 (0.01)	333.9 (2.6)	0.63 (0.02)
6. Insulin resistance	Sex, BMI (log), HOMA-IR	714.7 (702.3)	0.68 (0.01)	355.2 (5.0)	0.66 (0.03)	733.1 (0.2)	0.62 (0.00)	318.7 (0.7)	0.62 (0.01)
7. Hypertension	Sex, BMI (log), systolic BP, diastolic BP	722.4 (4.4)	0.68 (0.01)	361.0 (4.8)	0.66 (0.03)	738.7 (0.1)	0.63 (0.00)	324.6 (0.1)	0.63 (0.00)
8.Cardiometabolic	Sex, BMI (log), triglycerides(log), cholesterol, LDL-C, VLDL-C, HDL-C, HOMA-IR, systolic BP, diastolic BP	751.4 (8.0)	0.73 (0.02)	379.7 (12.5)	0.76 (0.04)	769.9 (2.4)	0.65 (0.01)	351.1 (2.7)	0.67 (0.02)
9. Liver enzymes	Sex, BMI (log), ALT (log), AST (log), GGT (log)	713.9 (9.3)	0.73 (0.02)	351.7 (10.4)	0.74 (0.04)	732.6 (2.7)	0.69 (0.01)	329.8 (1.8)	0.63(0.01)
10. Cardiometabolic and liver enzymes	Sex, BMI (log), WCHt ratio, triglycerides(log), cholesterol, LDL-C, VLDL-C, HDL-C, HOMA-IR, systolic BP, diastolic BP, ALT (log), AST (log), GGT (log)	752.8 (11.2)	0.77 (0.02)	374.0 (16.2)	0.84 (0.03)	776.1 (4.6)	0.73 (0.01)	373.4 (3.7)	0.70 (0.02)
11. Routine clinical measures	Sex, BMI (log), WCHt ratio, steatosis ^†^, triglycerides(log), cholesterol, LDL-C, VLDL-C, HDL-C, HOMA-IR, systolic BP, diastolic BP, ALT (log), AST (log), GGT (log)	764.4 (10.1)	0.78 (0.02)	381.5 (15.5)	0.84 (0.03)	783.0 (4.6)	0.73 (0.01)	380.3 (3.8)	0.70 (0.02)

(See Table 5) Amongst participants with a valid liver stiffness measurement, 1163 participants (59.4% female) had a BMI ≥ 25 kg/m^2^ in the 24-year clinic, whilst 1160 participants (64.6% female) participants had an overweight or obese BMI SDS in the 17-year clinic. At the 24-year the “cardiometabolic and liver enzyme” (model 10 AUROC 0.70 [SD 0.02]; BIC 373.36 [SD 3.72]) and “RCM” models (model 11 AUROC 0.70 [SD 0.01]; BIC 380.31 [SD 3.80]) again had the highest AUROCs. This was also the case at the 17-year time point (model 10 AUROC 0.84 [SD 0.03] and BIC 373.98 [SD 16.23]; model 11 AUROC 0.84 [SD 0.03] and BIC 381.49 [SD 15.54]). However, the “BMI” model had the lowest BIC at both the 24-year (model 2 BIC 312.42 [SD 0.00]; AUROC 0.62 [SD 0.00]) and 17-year time point (BIC 350.03; [SD 3.91]; AUROC 0.64 [SD 0.02]). At 17-years, the “steatosis” model also had a low BIC (BIC 350.53 [SD 7.56]; AUROC 0.67 [SD 0.04]).

### Decision curve analysis—NAFLD fibrosis at 24 years

**Fig. 2:**
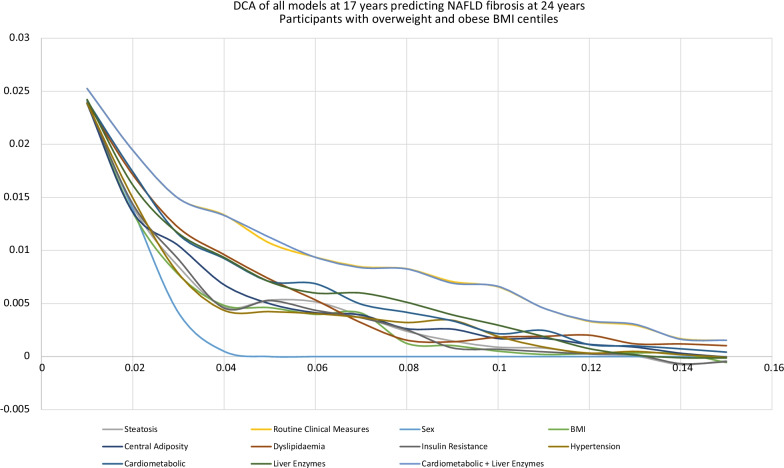
Decision curve analysis of different models to predict NAFLD Fibrosis at 24 years in 17 years with overweight or obese BMI SDS centiles

At the 17-year time point, in participants who had an overweight or obese BMI SDS centile, a decision curve analysis (DCA) was performed to inform which of the models presented thus far would help clinicians decide who to monitor for signs of early development of NAFLD fibrosis in young adulthood. A risk threshold of 0.1 (10%) was set i.e., we would recommend ongoing monitoring and management if the risk of fibrosis aged 24 years was 10% or more. Using a threshold of 0.1, the use of any model was superior to monitoring all patients with a “Treat All patients with an overweight BMI SDS” assumption (see Fig. 2). The model with the highest was the “RCM” model, with a median net benefit (NB) was 0.0054 (IQR 0.0034–0.0074). This was followed by the “cardiometabolic and liver enzymes” model (NB 0.0052 (IQR 0.0033–0.0070)) and then the “liver enzymes” model (NB 0.0019 (IQR 0.0012–0.0029)) (see Table 6).

**Table 6:** Net Benefit for different models at 17-year time point in participants with overweight/obese BMI SDS and all participants, predicting NAFLD fibrosis outcomes at 24 years

Model	Net benefit at risk threshold of 0.1 (median (IQR))	
	Participants with overweight/obese BMI SDS	All participants
Treat All	− 0.0748 (− 0.0858 to − 0.0620)	− 0.0841 (− 0.0963 to − 0.0722)
Treat None	0 (0)	0 (0)
Routine clinical measures	0.0054 (0.0034 to 0.0074)	0.0012 (0.0009 to 0.0019)
Cardiometabolic factors and Liver enzymes	0.0052 (0.0033 to 0.0070)	− 0.0003 (− 0.0006 to − 0.0005)
Liver enzymes	0.0019 (0.0012 to 0.0029)	0.0000 (− 0.0002 to 0.0003)
Cardiometabolic factors	0.0015 (0.0023 to 0.0052)	0.0006 (0.0002 to 0.0009)
Dyslipidaemia	0.0010 (− 0.0002 to 0.0018)	0.0004 (0.0001 to 0.0009)
Central adiposity	0.0001 (− 0.0004 to 0.0007)	0.0001 (− 0.0004 to 0.0007)
Insulin Resistance	0.0004 (− 0.0002 to 0.0008)	− 0.0002 (− 0.0004 to 0.0001)
Hypertension	0.0008 (0.0004 to 0.0015)	− 0.0001 (− 0.0003 to 0.0001)
BMI	− 0.0001 (− 0.0001 to − 0.0008)	− 0.0002 (− 0.0004 to − 0.0000)
BMI and steatosis	− 0.0001(− 0.0009 to 0.0011)	− 0.0003 (− 0.0005 to 0.0001)

Based on this decision curve analysis using a pre-determined threshold probability of 0.1 means these models will result in a net of 10 true positives (NAFLD) fibrosis per 100 patients. The “RCM” model would allow 5.4 fewer false positives (not NAFLD fibrosis) per 1000 patients, compared to 5.2 fewer false positives in the cardiometabolic and liver enzyme model. In contrast, the central adiposity, insulin resistance, hypertension and BMI models would not reduce false positives and therefore provide no net benefit. The difference of NB between the “RCM” model and the “BMI” model was 0.0054 to − 0.0001 = 0.0055. This is the equivalent to 5 more NAFLD fibrosis cases being detected per 1000 patients, for the same number of unnecessary referrals—i.e., cases that would not have developed into NAFLD fibrosis.

A net benefit of the different models was evaluated in all participants, equivalent to the general population, at the 17-year time point assessing NAFLD fibrosis (see Table 6). The “RCM” model had the highest net benefit, but this was small (NB 0.0012 (IQR 0.0009–0.0019), the equivalent of 1 more NAFLD fibrosis case being detected per 1000 patients treated.

## Discussion

### Main findings

Central adiposity, amongst overweight/obese participants in adolescence, had the greatest explanatory power for understanding future NAFL risk at 24 years. This was judged by BIC which we used to penalise model complexity. Central adiposity is a hallmark of the metabolic syndrome and has been repeatedly demonstrated to be associated with NAFLD development [27–29]. Interestingly, amongst all participants at adolescence, the “Insulin Resistance” model (containing HOMA-IR, BMI and sex) had the lowest BIC when examining future NAFL risk at 24 years. Insulin resistance is heavily implicated in NAFLD pathogenesis. It impairs insulin function to suppress lipolysis in adipose tissue, increasing free fatty acid delivery to the liver [30]. Insulin resistance, in combination with dyslipidaemia and the subsequent inflammatory cytokine milieu, which includes reductions in adiponectin and increased TNFα, culiminates in hepatic lipogenesis [31, 32]. Whilst these mechanisms occur simultaneously, insulin resistance appears be one of the earliest signals to detect in when considering future NAFL risk amongst adolescents.

When predicting NAFLD fibrosis at 24 years, adolescent BMI, in all participants and in those that were overweight/obese, had the greatest explanatory power for predicting this outcome, based on BIC. This was closely followed by the “Central adiposity” model, based on WCHt ratio, and "Steatosis” model based on ultrasound. BMI is often considered a crude measure to assess metabolic risk, with different BMI cut offs now suggested for obesity based on type 2 diabetes risk [33]. However, when considering future fibrosis risk, which confers increased liver related events and mortality, it remains the best marker within our cohort. This would need validation in another cohort, ideally amongst a non-Caucasian population.

Whilst BIC is a valuable tool to infer prediction of a future event, it cannot necessarily comment on the amount of true positive cases a model will generate. To clarify this further and make more relatable to clinic utility, we used a decision curve analysis exploring the net benefit of each model compared to treating all patients or treating none when considering the outcome of NAFLD fibrosis. What this demonstrated was that the “routine clinical measures” model was associated with the greatest net benefit above all the other models, at the pre-defined risk threshold probability of 10%. However, the net benefit was modest at only additional 5 more NAFLD fibrosis cases per 1000 patients.

As NAFLD fibrosis is poorly characterised in young adults, this study provides an insight into the clinical value of tools we have available in predicting patients that are going on to develop fibrosis in weight management clinics or the primary care setting. Unlike NAFL, it is fibrosis that is associated with liver related mortality [5].

### Strengths and limitations

Utilising a prospective longitudinal birth cohort study, this is one of the first attempts to explore how we can predict fibrosis burden from NAFLD amongst young adults. Within the ALSPAC cohort at 24 years, 2.7% had ≥ F2 equivalent fibrosis [8]. This however could be an underestimate of true population burden. Zhang and colleagues, using transient elastography on National Health and Nutrition Examination Survey (NHANES) participant, found the prevalence of ≥ F2 fibrosis to be 16.7% in 20–29 year olds [34]. This can in part be explained by the demographic profile of ALSPAC and its catchment area population in the South West. Differential attrition has created an overrepresentation of more affluent groups and underrepresentation of ethnic minorities [10]. Furthermore, the South West UK region has the 3^rd^ lowest adult prevalence of obesity in UK, at 23%. Therefore, this study may underestimate young adult NAFLD prevalence in the whole UK, particularly regions such as the North East UK, where obesity adult prevalence is 30%.

This study centred around prediction of future risk of NAFLD fibrosis in adolescence. The “BMI” and “central adiposity” models had the best explanatory power for understanding future risk, however we do not have data on inter- and intra-observer variability of these measurements within the Teen Focus 4 clinic. If there was substantial variability, this would bias our estimates and models overall.

Future work is required to validate our findings in other general population studies, particularly in other ethnic groups, to assess what tests are helpful to evaluate NAFLD fibrosis risk specifically in young adulthood. This study has also not commented on test trade-off, as routine blood tests are inexpensive and relatively non-invasive. Assessment of models such as this in bariatric populations that may require surgery would benefit from additional harm and cost trade-off analyses when considering the outcome of NAFLD fibrosis in young adults.

Whilst transient elastography has good receiver operator characteristics for the detection of fibrosis, we are unable to comment on non-alcoholic steatohepatitis outcomes in our 24-year clinic as biopsy was not feasible in a general population study and the clinic did not have access to magnetic resonance elastography (MRE)/spectroscopy (MRS). As drug trials for the treatment of NASH and fibrosis develop and become more accessible, the net benefit generated from a low-risk threshold may rise.

### Other evidence

Our evaluation of predicted risk of NAFL and fibrosis conflict previous work performed in older adults with NAFLD, where type 2 diabetes/insulin resistance has been demonstrated to be the best predictor of fibrosis risk. Park et al. performed MRE on 2149 participants (mean age 50 years) within the Kangbuk Samsung Health Study, a general population study, having excluded participants with viral hepatitis, harmful alcohol consumption and cirrhosis [35]. Significant fibrosis (≥ F2 fibrosis) prevalence was 3.9% in this group. Five models were compared including participants with ultrasound confirmed steatosis, elevated liver enzymes, the metabolic syndrome, impaired fasting glucose and type 2 diabetes, with the type 2 diabetes group having the best receiver operator characteristics for detection of NAFLD fibrosis (≥ F2) and the net benefit of 0.0075 at a threshold probability of 0.1 [35]. We were unable to comment on type 2 diabetes risk as to the best of our knowledge, none of our participants were known to have type 2 diabetes aged 17 years. In adolescence our finding was that BMI remained the strongest predictor of NAFLD fibrosis, which could indicate mechanistically different risk factors have more prominent effects at different times in the life course.

Bedogni et al. examined models exploring steatosis (not fibrosis) risk in adolescents with obesity (mean age 15 years), comparing BMI with ALT, HOMA-IR, triglycerides, and uric acid to a second model replacing BMI with waist circumference and keeping the other listed variables [36]. When assessing adolescent NAFL at this time point the BIC for the BMI model was marginally better than the waist circumference (a surrogate for central adiposity) model [36]. The same group examined risk factors for steatosis, due to NAFLD and alcohol, in older adults using the Bagnacavallo Study. The model which generated the lowest BIC was one which included liver function tests, sex, age, BMI, and alcohol intake. This was superior to other models that also included waist circumference, lipid profile, BP, and serum glucose [36]. Whilst we specifically presented prediction models excluding those with harmful alcohol consumption, in the general population setting we found our “central adiposity” model (trunk fat mass, BMI and sex) to be the best predictor of steatosis. In supplementary analysis including all participants regardless of alcohol consumption, we did not find alcohol consumption (as an ordinal variable) to improve the BIC over and above central adiposity (see Additional file 1).

### Implications

Future work is required to validate our findings in other general population studies to assess how we can best assess adolescents for risk of developing NAFL and NAFLD fibrosis in the general population setting and in obesity clinics. Central adiposity and BMI in adolescence were consistently found to be the best predictors for future NAFL and fibrosis respectively. These are clearly major drivers for NAFLD development, and it could be argued focusing management on these risk factors could prevent future harm from fibrosis, through weight management support primarily. It has been demonstrated that a weight reduction of 5–7% is associated with a reversal of NAFL and non-alcoholic steatohepatitis [37], and weight loss of ≥ 10% is associated with hepatic fibrosis reversal [38]. This is perhaps best illustrated in the bariatric population where lasting NASH resolution and absence of fibrosis progression has been demonstrated at 5 years post-operatively [39]. Access to weight management services in patients who are unable to meet their weight reduction is notoriously mixed, with long waiting lists across the UK. This needs to be addressed and discussed nationally. At a patient level, intensive weight management has ubiquitous benefits and can be long lasting, even in underserved populations. A randomised controlled trial comparing an 18 month monthly intensive-lifestyle program to standard of care showed an almost 5% sustained weight loss at 2 years [40]. In patients with type 2 diabetes with NAFLD, the threshold for glucagon-like peptide-1 analogues such as Liraglutide and Semaglutide, which are associated with weight loss and NASH resolution, needs to be lower [41, 42]. At a population level, the financial incentives are clear, with the NHS spending £6.1bllion on overweight and obesity-related-ill health and obesity costing the wider UK economy approximately £27 billion annually [43]. The UK produced a Prevention Green Paper in 2019 and Policies such the “Soft Drinks Industry Levy” introduced in the UK Prevention Green Paper in 2019 have had positive effects in reducing consumption of sugary drinks [44, 45].

## Conclusion

Central adiposity and BMI in adolescence are the best predictor of NAFLD fibrosis development in young adulthood. Additional measurement of cardiometabolic factors such a lipid profile, liver blood tests, HOMA-IR, blood pressure, do provide additional net benefit in detecting true positive fibrosis cases but this benefit is small. Thus, the agenda of reducing morbidity and mortality associated with NASH cirrhosis, the ultimate end point of NAFLD, the focus must be on obesity. Tackling obesity is a global effort that at a local level involves improving access to weight management services but more holistically requires an acknowledgement that the obesogenic environment we live in must be altered rather than relying on individuals to make personal choices about diet and exercise.

## Supplementary Information

**Additional file 1.** **Supplementary Table 1.** Summary of models auditioned at 17-years, including alcohol consumption, with fibrosis at the 24 years. **Supplementary Table 2.** BIC and AUROC at 17-year timepoint assessing the outcome of fibrosis including alcohol consumption at 24 years (n=3599). Imputed results presented.

## Data Availability

The data that support the findings of this study are available from ALSPAC but restrictions apply to the availability of these data, which were used under license for the current study, and so are not publicly available. Data are however available from the authors upon reasonable request and with permission of ALSPAC Executive committee (see http://www.bristol.ac.uk/alspac/researchers/access/).

## Abbreviations

ALSPAC: Avon Longitudinal Study of Parents and Children
ALT: Alanine transaminase
AST: Aspartate aminotransferase
AUD: Alcohol use disorder
AUDIT: Alcohol Use Disorder Identification Test
AUROC: Area under the receiver operator curve
BIC: Bayesian information criterion
BMI: Body mass index
CAP: Controlled attenuation parameter
DSM-V: Diagnostic and Statistical Manual of Mental Disorders V
F@24+: Focus at 24+ clinic
GGT: γ-Glutamyl transferase
HDL-C: High density lipoprotein-cholesterol
HOMA-IR: Homeostasis Model Assessment—Insulin Resistance
IQR/M: Interquartile range/median ratio
LDL-C: Low density lipoprotein-cholesterol
LFTs: Liver function tests
LSM: Liver stiffness measurement
MRI PDFF: Magnetic resonance imaging proton density fat fraction
NAFL: Non-alcoholic fatty liver
NAFLD: Non-alcoholic fatty liver disease
NASH: Non-alcoholic steatohepatitis
RCM: Routine clinical measures
ROC: Receiver operator curve
TE: Transient elastography
TF4: Teen Focus 4 clinic
USS: Ultrasound scan
VLDL-C: Very low density lipoprotein-cholesterol
WCHt: Waist circumference-to-height
