# Supplementary material for: Evaluating future risk of NAFLD in adolescents: a prediction and decision curve analysis
Source: BMC Gastroenterol. 2022 Jun 30;22:323. doi: 10.1186/s12876-022-02401-y (PMC9245230; doi:10.1186/s12876-022-02401-y)
Supplement: Supplementary file 1 — Additional file 1. Supplementary Table 1. Summary of models auditioned at 17-years, including alcohol consumption, with fibrosis at the 24 years. Supplementary Table 2. BIC and AUROC at 17-year timepoint assessing the outcome of fibrosis including alcohol consumption at 24 years (n=3599). Imputed results presented. [file 12876_2022_2401_MOESM1_ESM.docx]

**Title:** Evaluating future risk of NAFLD in adolescents: a prediction and decision curve analysis.

**Authors:** Kushala W M Abeysekera^1, 2^ *, James G Orr ^2^, Fiona H Gordon ^2^, Laura D Howe ^1,3^, Julian Hamilton-Shield ^4^, Jon Heron ^1^, Matthew Hickman ^1, 3^

**Affiliations:**

1 – Population Health Science, Bristol Medical School, University of Bristol, Bristol, UK.

2 – Department of Liver Medicine, University Hospitals Bristol and Weston NHS Foundation Trust, UK

3 – MRC Integrative Epidemiology Unit, University of Bristol, Bristol, UK

4 – NIHR Bristol Biomedical Research Centre, Nutrition Theme, University of Bristol, Bristol, UK

**Corresponding author address:**

Population Health Sciences

Oakfield House

Oakfield Grove

Bristol BS8 2BN

Email address: [k.abeysekera@bristol.ac.uk](mailto:k.abeysekera@bristol.ac.uk)

**Key words:**

ALSPAC (Avon Longitudinal Study of Parents and Children); Body composition; NAFLD (nonalcoholc fatty liver disease); obesity; young adults.

**Supplementary material**

**Supplementary methods**

We examined all the participants with available CAP and liver stiffness measurements regardless of alcohol intake, to explore whether alcohol consumption improved the prediction of the outcomes of fibrosis overall in all adolescent participants in the Teen Focus 4 clinic. Alcohol consumption was stratified using a 3-category ordinal variable separating low risk drinkers (AUDIT-C score < 5 and absence of AUD), hazardous drinkers (AUDIT-C score of 5+ and absence of AUD) and harmful drinkers (evidence of AUD).

Supplementary Table 1. Summary of models auditioned at 17-years, including alcohol consumption, with fibrosis at the 24 years

| Model | Model name | Model components at 17 years |
| --- | --- | --- |
| 1 | Sex | Sex |
| 2 | BMI | Sex, BMI (log) |
| 3 | BMI + alcohol | Sex, BMI (log), alcohol use |
| 4 | Central adiposity+ alcohol | Sex, BMI (log), WCHt ratio, alcohol use |
| 5 | Steatosis + alcohol | Sex, BMI (log), USS defined steatosis, alcohol use |
| 6 | Dyslipidaemia+ alcohol | Sex, BMI (log), triglycerides(log), cholesterol, LDL-C, VLDL-C, HDL-C, alcohol use |
| 7 | Insulin resistance+ alcohol | Sex, BMI (log), HOMA-IR, alcohol use |
| 8 | Hypertension | Sex, BMI (log), systolic BP, diastolic BP, alcohol use |
| 9 | Cardiometabolic+ alcohol | Sex, BMI (log), triglycerides(log), cholesterol, LDL-C, VLDL-C, HDL-C, HOMA-IR, systolic BP, diastolic BP, alcohol use |
| 10 | Liver enzymes+ alcohol | Sex, BMI (log), ALT (log), AST (log), GGT (log), alcohol use |
| 11 | Cardiometabolic, liver enzymes, alcohol | Sex, BMI (log), WCHt ratio, triglycerides(log), cholesterol, LDL-C, VLDL-C, HDL-C, HOMA-IR, systolic BP, diastolic BP, ALT (log), AST (log), GGT (log), alcohol use |
| 12 | All | Sex, BMI (log), WCHt ratio, ultrasound defined steatosis, triglycerides(log), cholesterol, LDL-C, VLDL-C, HDL-C, HOMA-IR, systolic BP, diastolic BP, ALT (log), AST (log), GGT (log), alcohol use |

**Supplementary Results**

3599 participants had valid liver stiffness measurements from the Focus@24 clinic (mean LSM 4.7kPa; SD 1.5). The predictive ability of models to detect fibrosis, regardless of cause, was examined at 17 years with the addition of alcohol consumption in all participants. (see supplementary Table 2) This did not alter results seen in models predicting NAFLD fibrosis, with the “All” model having the highest AUROC (model 12 AUROC 0.77 SD [0.02]; BIC 926.68 [SD 11.41]) , but the lowest BIC was again seen in the BMI model (model 2 AUROC 0.68 [SD 0.01]; BIC 864.25 [SD 3.70]).

| Model | 17-year model | |
| --- | --- | --- |
|  | All participants  (n=3599) | |
|  | BIC  Mean (SD) | AUROC  Mean (SD) |
| 1. Sex | 889.1 (0.8) | 0.59 (0.00) |
| 2. BMI | 864.2 (3.7) | 0.68 (0.01) |
| 3. BMI + alcohol | 872.3 (3.7) | 0.68 (0.01) |
| 3. Central adiposity + alcohol | 876.6 (3.9) | 0.68 (0.01) |
| 4. Steatosis + alcohol | 877.0 (6.3) ^†^ | 0.68 (0.01) |
| 5. Dyslipidaemia + alcohol | 900.3 (7.0) | 0.70 (0.01) |
| 6. Insulin resistance + alcohol | 868.6 (5.3) | 0.68 (0.01) |
| 7. Hypertension + alcohol | 886.6 (3.9) | 0.68 (0.01) |
| 8.Cardiometabolic + alcohol | 918.5 (8.1) | 0.71 (0.01) |
| 9. Liver enzymes + alcohol | 878.4 (6.4) | 0.72 (0.01) |
| 10. Cardiometabolic and liver enzymes + alcohol | 918.3 (10.2) | 0.76 (0.02) |
| 11. All variables | 926.7 (11.4) | 0.77 (0.02) |

Supplementary Table 2. BIC and AUROC at 17-year timepoint assessing the outcome of fibrosis including alcohol consumption at 24 years (n=3599). Imputed results presented.

**Declarations**

**Ethics approval and consent to participate**

Ethical approval for the study was obtained from the ALSPAC Ethics and Law Committee and the Local Research Ethics Committees. Consent for biological samples has been collected in accordance with the Human Tissue Act (2004). Informed consent for use of data collected via questionnaires and clinics was obtained from all participants following the recommendations of the ALSPAC Ethics and Law Committee at the time. The study was carried out in relevant guidelines and regulations.

**Consent for publication**

Not applicable in the declarations section.

**Competing interests**

The authors declare that they have no competing interests.
